# On Reflex Motions

**Published:** 1838-07-01

**Authors:** 


### ON REFLEX MOTIONS. By Dr. *A. W. Volkmann*, Professor of Physiology in Dorpat.

[From the Archiv Für Anatomie, Physiologie, by Dr. J. Müller, 1838.]*

BY the term "Reflex Function" Marshall Hall has, it is well known, characterized an action of the spinal cord, which imparts muscular motions not in the direct way of nervous communication, but in a round-about way. The round-about consists in this, that the exciting stimulus affects a peripheric organ, as, for instance, the skin is carried from thence to the spinal cord; and from this it is at length reflected to the muscles which are to be moved. Phenomena which appertain to the reflex functions, were well known a long time since, as may be proved from the works of Le Gallois, Desmoulins, Flourens, and Treviranus, yet Marshall Hall has the merit of having investigated them more fully, and of having compared them with nervous actions of quite another kind. Though I duly appreciate this merit, and wish to do full justice to the important discoveries of the English physician, still I cannot deny that I consider many of the consequences which he has deduced from his experiments as not being sufficiently grounded, nay even as being absolutely erroneous.

The chief points in the theory laid down by Dr. M. Hall are as follows: The reflex motions of the spinal cord make a *receptive* and a *re-active* faculty necessary in the latter, but the receptive is not *sensation*, nor is the re-active the will. As according to Bell's discovery, sensation and will have their own nervous fibres, so have both these faculties of the spinal cord in like manner their own proper fibres, which are not connected with the sensorium, but solely with the spinal marrow. The fibres subservient to the receptive faculty are called exciting, those appertaining to the re-active faculty are styled excito-motory fibres. Now as the fibres subservient to sensation and those to motion enter together into the mixed nerves, so is there again a more complex mixture which proceeds from the combination of the senso-motory nerves with the excito-motory. Through the reflex functions of the spinal cord the sphincters are closed, and the tone of the muscles is preserved. The reflex motions are just as distinct from the voluntary motions, as from the motions of muscular irritability, and form accordingly a class of motions of a peculiar kind.

The extraordinary importance of the reflex functions in physiology and practical medicine, induced me to communicate the following article, in which I speak entirely from my own experience, though for the sake of connexion I deem it necessary to make some observations which are already known from the works of Dr. M. Hall and John Müller.

### 1.—STATE OF FROGS AFTER DECAPITATION.

If the spine of a frog be divided perfectly behind the skull, violent motions always take place in the trunk, which hold for a little time, and cease in a variety of ways. Very frequently there is observed so strong an adduction

---

* Contrary to our usual custom, we have deemed it necessary, in this instance, to give rather a translation than an analysis, in order to prevent any mistake or misapprehension, where nice physiological experiments are narrated.

of the hind legs, that the feet are extended forward over the head, in consequence of which motion the body sometimes completely tumbles over. In other cases the effect of the decapitation is directly the contrary, as a violent extension of the hind-legs, combined with rigidity takes place, in which case a rooting (wühlende) motion of the muscles of the extremities is observed. On what peculiar circumstances this variety of the motions depends, I have not been able to ascertain. Marshall Hall's assertion is of physiological importance, viz. that in the decapitated animal, if it once comes to be at rest, motions never again take place in it, unless external irritants occasion them. The decapitated animal must continue in the state of rest once assumed, and remain unchangeable till the last spark of life disappears. Now I can most positively state that this assertion is incorrect. I have repeatedly seen that decapitated frogs, without any external cause whatever, made certain motions with the posterior extremities, apparently, as if the animal wished to place itself in a commodious position. I can even mention a certain way by which to observe similar independent motions in decapitated frogs. If the head be separated from the trunk, and the first spasmodic movements be over, a state of rest takes place, which appears to be a consequence of exhaustion. At this period, commonly a few minutes after decapitation, the mutilated body is very little excitable, and whilst at a later period the slightest touching of the skin occasions reflex-motions, the body may now be handled in a variety of ways, without causing any motions. If at this period one brings the posterior extremities into a perfectly extended position, and allows the animal to lie at rest on the solid ground, it will be observed, that this position will continue from five to ten minutes, but afterwards the frog adducts the legs, not gradually, but suddenly, and changes his extended, lying position for a sitting one. I do not remember that this experiment ever even once failed me. But if the frog has at first assumed the sitting position, he commonly continues in it till death, and an independent movement is seldom observable in the absence of external stimuli.

### II.—Simplest Phenomenon of Reflex Motions.

On irritating a decapitated amphibious animal by pricking, pinching, burning, often also by merely feeling with the hand, the animal makes regular muscular motions, which oftentimes perfectly resemble those of voluntary motion. These motions do not consist merely of a simple and sudden start, as occurs when a muscle is irritated, but they are more extended as to time, and complicated with respect to their consequences. The motions are repeated for example in the alternation of flexion and extension, particularly if the first motion, by striking the limb against some hard object, becomes itself a stimulus for a second motion. Continued reflex motions are also observed, when the amphibious animal is suspended, so that the continuance of the motion must be referred to the uninterrupted action of an internal principle. These phenomena continue in lively frogs, salamanders and water serpents, always for several, often for very many hours after death, they cease on the contrary instantaneously when the spinal marrow is destroyed or divided. If an amphibious animal be divided into several portions, these reflex motions are observable in every part furnished with spinal marrow, and cease in each the moment the spinal marrow is destroyed.

### III.—Reflex-motions on Dividing the Spinal-cord Longitudinally.

In a decapitated frog, the upper half of the spine was opened, and a deep longitudinal cut was carefully made from the commencement of the spinal marrow to two lines below the second spinal nerve, and so on to the fourth nerve of the spine. In consequence of this operation, an uninterrupted spasmodic movement took place in the muscles of the anterior extremities, and slight twitches in the muscles of the thighs. When this storm was allayed, the animal assumed a sitting posture. When I now touched one of the fore-paws, this immediately drew itself back. When on the contrary I pinched one fore-paw with a pincers, not only the irritated paw moved itself, but both hind-legs drew back violently. On each attempt at irritation these phenomena were repeated, on the contrary I never remarked that pinching a fore-paw occasioned any motions in the corresponding one of the other side.

On irritating one hind-leg in the same animal, not only were reflex motions occasioned in the second hind-leg and in the fore-paw of the same side, but to all appearance convulsive movements in the fore-paw of the opposite side. On pinching the left hind-leg, the animal wheeled round, with the thorax to the left-side, which could not take place otherwise than by stretching the right shoulder. I could now decidedly observe twitches in the muscles of the shoulder, which however also receive their nerves from the part of the spinal-cord which was divided longitudinally.

In a decapitated frog the spine was opened in the lumbar region, and that portion of the spinal marrow divided by a longitudinal cut, which supplied the posterior half of the body with nerves. I now irritated one fore-paw, when all the four extremities moved every time quite perceptibly. If on the contrary I irritated a hind-foot, reflex motions were observed only in the fore-foot of the corresponding side. They were of a doubtful nature in the fore-foot of the opposite side, whilst in the second hind-foot no trace of motion was observable.

In another frog, the spine was opened along its entire length, and the spinal marrow was split longitudinally from the upper extremity to the last lumbar nerve. The reflex motions were very feeble. Irritation of the anterior extremities could never produce motions in the posterior, a thing which was obviously the consequence of the too great destruction of parts. Irritation of a hind-foot produced motions in the corresponding leg and abdominal muscles of the same side. Severe pinching in the same even excited twitchings in the m. cucullaris of the same half of the body. On the contrary, twitchings were never occasioned in muscles of the opposite side, though the portion of the spinal marrow which lies behind the roots of the sciatic plexus had still continued uninjured.

In a decapitated frog the spine was opened, with the exception of the fourth and fifth vertebræ, and the spinal-marrow was halved along its entire length, except the place covered by the vertebræ just mentioned. Accordingly only a small portion of the spinal-marrow remained undivided—that which supplies the middle abdominal region with nerves. Irritating one fore-paw merely excited motions in this part itself, whilst irritation of a hind-foot, on the contrary, brought reflex motions in all the four extremities.

In this case the motion of both shoulders was very striking, that of the arm distinct only on the same side with the irritated leg.

In the preceding observations the strength of proof lies only in the cases where a motion follows, not in those where it ceases. For it is clear that a reflex motion can only take place when its organic conditions are given, whilst in operations such as the preceding, it must very frequently cease from the knife having been too destructive. With respect to this circumstance, I think I am warranted in concluding from all that is premised: *that a longitudinal division of the spinal marrow does not prevent the extension of reflex motions over all the muscles of both halves of the body, so long as any part of the proper spinal-marrow remains connected at the middle line.* By proper spinal-marrow I mean that part from which the first ten spinal nerves take their origin. The portion of the spinal-marrow lying more posteriorly, which part might be called the pars caudalis, seems to be incapable of reflecting irritation from one side of the body to the other.

### IV.—Reflex Motions have the Character of Determinateness (Zweckmässigkeit).

When a decapitated amphibious animal is irritated, the motions consequent on the irritation are not only generally determinate, in as far as the muscles associated during life come into action simultaneously, whilst the antagonist muscles, on the contrary, act in fixed sequence, but they are also especially determinate, that is, the re-action of the motion depends on the peculiar mode of irritation. If one stimulates a decapitated tortoise, it crouches beneath its shell. It is a striking thing that frogs, which are not accustomed to those peculiar movements, prefer them after decapitation. It is equally striking that these peculiar movements are not always performed in the same way, but in a way varying very much according to circumstances. I irritated the fore-paws of a decapitated frog, and it drew them back. I irritated them again, and again he drew them farther back; but on irritating them once more, the animal hid its paws under the abdomen, and changed its sitting into a lying posture. If one stimulates a decapitated frog in a sitting posture severely on one hind-leg, he makes a spring, and so extends the thigh; if one grasps him roughly in the pectoral region, he stretches the thigh forwards, presses his feet firmly against the hand, which holds him, and seeks to free himself. If a person pinches the skin of the abdomen or spine with a pincers, nothing is more common than that the mutilated animal should scratch the irritated part with the hind-leg of the corresponding side.

It appears on the whole, as if the decapitated animal felt the operation of the stimulus, and from among various means selected the most suitable to remove itself from the trouble of so feeling.

### V.—The Extension of the Reflex Motions is principally dependent on the Strength of the Stimulus and on the Degree of Irritability.

When a person irritates a recently decapitated amphibious animal by gently touching any part, the motion is often confined to the vicinity of the part irritated. Thus, by gently irritating a toe, it sometimes happens that motions of the foot exclusively are produced. But by a somewhat stronger irritation the entire limb, of which a part is touched, becomes moved; by increasing the irritation still farther, the motions extend over all the muscles, and it appears worthy of remark, that proportionally slight irritations, for instance, gentle feeling with the hand, are strong enough to produce general motions.

In a corresponding way the extension of the reflex motions depends on the degree of irritability. The less the irritability, the more confined the movements are, the intensity of the irritation in other respects continuing the same. Thus a considerable time after decapitation it is no longer possible by irritating a limb to produce motions in other parts except in those irritated.

### VI.—In the Reflex Functions the Posterior Roots of the Spinal Nerves serve exclusively as exciting, the Anterior exclusively as reflecting Nerves.

By exciting nerves, I mean such as are capable of conducting the irritation, which is applied to a peripheric organ, inwardly to the spinal-marrow, and by reflecting nerves, those which conduct the irritation from the spinal-marrow outwards from the centre, and convey it to a muscle.

If in a decapitated frog the three posterior roots of the sciatic plexus be cut through, the most violent irritation of the thigh is not able to produce reflex motions. But it can be shown that this same operation has neither destroyed the reflecting power of the spinal cord, nor the muscular irritability of the injured thigh. If, for instance, in the same frog, one irritates a fore-paw, reflex motions are observable in all the four extremities. If then dividing the posterior roots of the sciatic plexus deprives the divided nerves of the power of producing reflex motions, this circumstance can only depend on the posterior roots alone being qualified to convey a peripheric irritation to the spinal-marrow.

If in a decapitated frog the anterior roots only of the crural plexus be divided, on irritating the affected leg, no motions are produced in the limb itself, but motions are produced in the three remaining extremities. If in the same frog any one of the uninjured extremities be irritated, reflex motions are excited in all the muscles with the exception of those of the extremity operated on. Accordingly the posterior roots of the spinal nerves contain no fibres, which possess the property of conducting an irritation from the spinal marrow in a centrifugal direction outwards, and of throwing it back to the muscles.

The experiments here communicated are only an imitation of those so skilfully performed by J. Müller (Physiologie Bd. 1. S. 703). However they have another confirmatory proof, as the latter. J. Müller operated on animals not decapitated, in which the motions of mental origin cannot be rigorously distinguished from the reflex motions. Animals not decapitated, when irritated, perform motions, which do not depend necessarily on the reflecting power of the spinal marrow, but possibly on the brain, in which sensation probably always comes into play. In non-decapitated animals also a motion sometimes does not follow, which in an animal deprived of brain would have taken place in consequence of a stimulus, and probably the reason is because the will has the power of interfering with the reflex motions.

Müller's experiments, strictly taken, only shew that the sensitive fibres are capable of an exclusive conducting inwards, whilst the voluntary motor fibres alone possess the property of conducting outwards. But if it be undetermined whether the sensitive and exciting fibres on the one hand, and the voluntary motor and reflecting fibres on the other hand, are identical (Dr. M. Hall has actually raised doubts with respect to this identity), experiments with decapitated animals were necessary in order to determine whether the posterior roots of the spinal nerves, besides sensibility, also preside over the exciting faculty, and whether the anterior roots, besides voluntary motion, also preside over reflex motion.

### VII.—The Efficacy of the Stimuli which produce Reflex Motions, is modified and increased through the Peripheric Distribution of the Nerves.

In a recently-decapitated frog the gentlest stimulus is sufficient to produce reflex motions, though the epidermis prevents an immediate action of the stimulus on the nervous mass. If on the contrary a nerve be laid bare, it becomes necessary to stimulate it with considerable strength, in order to produce reflex motions. In experimenting carefully every one will convince himself that no deception is present in this case. The preponderance of the irritability of the skin in frogs is most striking, after first narcotising them with opium and then decapitating them. Under these circumstances the irritability of the skin is sometimes encreased to such a degree, that tickling it with a feather produces general convulsions. If, in an animal so prepared, a piece of skin be removed, one may pinch and puncture the subjacent muscles, without occasioning any reflex motions whatever. Even dividing the muscles with the scissors at this period I have seen pass away without any reflex motions whatever.

Even without the previous exhibition of opium, the skin of the frog possesses a greater irritability than the nerves to which it is indebted for its power of sensation. On a decapitated animal I made the following experiment: I carried a circular incision with a scissors, commencing at the nape of the neck, over the left shoulder, the flanks, and the thigh of the left side, continuing it to the anus, and then carried it back on the right side through the corresponding regions. It is a known fact that in frogs the skin is very imperfectly connected with the rest of the body, and by the above-mentioned circular incision a considerable flap of skin was raised, which was connected with the remainder of the body only through some bands of cellular tissue, through blood-vessels and cutaneous nerves. This portion of skin, however, excited distinct reflex motions, whenever it was pinched with the pincers; more particularly stimuli in the region of the anus excited convulsive kicking with the hind feet.

The piece of skin was now held gently by the anterior edge, raised up, and separated from the body by cutting all the connecting fibres. In this operation, on making two cuts, slight, scarcely perceptible convulsive movements were occasioned on the upper part of the thigh, in the other parts no reflex motions. The experiment was repeated in the same animal towards the abdominal region. Irritating the flap of skin formed here by means of the pincers, not only produced very lively, but also determinate reflex motions : for if a part of the skin was pinched, the animal seized it with the fore-paw and covered it. I carefully removed this flap of skin with the scissors, and no reflex motion was produced.

On dividing larger nervous branches, or one of the posterior roots of the sciatic plexus, I have often observed reflex motions to be produced, but the character of determinateness, which is so prominent in cutaneous irritation, appeared invariably to be wanting. Were the animals on which I operated not decapitated, I would have admitted that irritation of the skin occasions objective conceptions* from such irritation, and accordingly a reflex action of the mind, whilst irritation of the nervous branches, on the contrary, produces only excitation of the spinal cord, and in consequence of this, a re-action which is organic to be sure, but still not mental.

Our author next considers the effects of irritating the sympathetic nerve, and the widely extended reflex motions occasioned thereby. He then proceeds to establish that the conveyance of the nervous principle from the periphery to the central organs, and from these backwards to the peripheric nervous distributions is not subject to the same laws, as its conveyance in the nerves of sensation and motion. He then endeavours to prove that our present experience is not sufficient to prove that all reflex motions of decapitated animals, and specifically of decapitated amphibious animals, can go on without the co-operation of the mind, as the principle of sensation and of the will.

M. Hall has excluded this co-operation of the mind for all cases, and the principal argument on which he rests, is the supposed experience that decapitated animals, if they are once in a state of rest, can perform no motions without the aid of an external stimulus, but that they die in the position once assumed. I have shown that this assertion is incorrect, so far at least as concerns stimuli, whose external nature is provable. I certainly will not absolutely deny that the motions which a decapitated animal at rest recommences independently, may still depend on something external, on some irritation of the air on the surface of a wound, and the like; but the admission of external stimuli of this kind would be mere hypothesis, and at the very least would leave behind the possibility that such motions really depend on an internal principle, which might even be a mental one.

Even if it were true that decapitated animals attempt no motions of themselves, M. Hall's conclusion would still be unsatisfactory, viz. that reflex motions are accomplished without the co-operation of the mind. Even where the system is uninjured, a state of mind exists in the period of profound sleep, where motions originally proceeding from the mind become interrupted.

During this period sensation and volition are not taken away, for they are both active on the individual awaking; they are only incapable of allowing actions to proceed from themselves without a preceding external stimulus.

---

* The English reader will excuse us for this scrap of metaphysical jargon; he has it however as we found it in the German, and every body knows what German metaphysics are.—*Reviewer.*

If it could be admitted that the mind may be contained in some other part of the body besides the brain, one might compare the rest of a decapitated animal to the rest of an animal asleep, with this difference however, that in the decapitated animal, where the rest was not caused by exhaustion, moderate stimuli rendered awaking from the torpor possible.

Extremely insufficient are the grounds by which M. Hall seeks to prove that sensation does not come into play in the reflex motions. He here remarks, that the reflex motions sometimes cease if the body be placed in a posture which must be incommodious, or even painful to the animal, if it felt. In a decapitated serpent the reflex motions ceased whilst the animal hung with its tail over the sharp edge of a table. It did not make a single move, when the tail was punctured, or even burned. It is strange that M. Hall did not explain these phenomena in the way in which alone they can be explained, viz. that the irritability of the subject was for the moment so exhausted, that the stimuli which were applied did not excite the action of the reflex functions. As puncturing and burning in decapitated animals in the normal state occasion motions, so the cessation of these motions, in the observed case, proves the cessation of a power which is ordinarily present. If in the individual case one deduces the loss of motion from the want of sensation, so is the latter generally recognized as a condition of motion, and vice versâ: if it be denied that the reflex motions proceed from sensation, the discontinuance of the one cannot prove the absence of the other in the single case. All those experiments which are intended to contradict the participations of the soul in the reflex motions are insufficient. If the observations be duly considered, which have been made by me regarding the determinateness of several reflex motions, it will be difficult to abandon the idea, that here also the mental principle comes into play. On all vital actions the character of determinateness is impressed, and yet all the vital functions do not proceed from the soul; still the determinateness of mental acts has a something peculiar, and many reflex motions seem to me to participate in this peculiarity.

The settling of the question, whether the determinate reflex motions of decapitated amphibious animals proceed from sensation and volition, would presuppose psychological investigations, to the prosecution of which I do not feel myself adequate; my end is attained if I have shown, that the phenomena already discussed are not sufficient to disprove the influence of the mind.

### VIII. On the Division of the Nerves, proposed by M. Hall.

M. Hall distinguishes two species of centripetal acting fibres, the *sensitive*, which are the mediators of sensation, and the *exciting*, which have nothing in common with sensation. In like manner he distinguishes two species of centrifugal acting fibres, the one of which produces only voluntary motions, *spontano-motory*, and the second of which also calls forth motions, but only involuntary motions (*reflecto-motory*). These four species of fibres are divided into two classes; the one comprehends the cerebral or senso-motory nerves, which are connected with the sensorium, the other contains the spinal or excito-motory nerves, which have no connexion with the sensorium. The nomenclature already indicates, and numerous passages in the essay on the nervous system scarcely leave a doubt, that M. Hall derives the reflex-functions exclusively from his spinal nerves, that is, from nerves whose fibres are not appropriated to the conducting of sensation and of the will. Many considerations, however, are opposed to this view of the subject.

If the reflex functions are made to proceed from the spinal nerves, a number of phenomena are struck out of the class of reflex-functions, which are essentially connected with them. In reality every voluntary motion is a reflected motion, as it proceeds from a conception which was brought into life through a sensible impression of some kind, and takes place by means of the brain as an intermediate organ transforming the received stimulus into a stimulus of the will, and transferring it to the spontano-motory fibres. No doubt, between these motions and the reflex motions in the strict sense, there is this distinction, that the former takes place through consciousness; but however important this may be in another respect, it seems totally indifferent with respect to the physiological act of reflection. Motions, as sneezing in consequence of a sharp smell, or contraction of the pupil by the stimulus of a strong light, could not be separated from reflex motions without considerable violence, and still in these cases the function evidently does not go on merely in the sphere of the spinal nerves, but with respect to the centripetal nervous conduction, by means of the cerebral nerves.

But even concerning the reflex functions in the stricter sense, it appears to me very doubtful, whether it is right to limit them to the excito-motory fibres, which are in no way connected with the organ of mind. One cannot touch even the smallest part of the skin with the finest needle, which does not possess sensation, from which we are warranted in concluding that every, even the smallest, part contains a sensitive nervous fibre. But if every prick with the finest needle excites reflex-motions in the decapitated frog, and if the excitation is to come entirely from spinal fibres, we are forced to the extraordinary admission, that every part of the skin, of the size of a needle's point, possesses two specifically distinct nervous fibres. For the same reason, every muscular fibre, which can be moved both voluntarily and also involuntarily, as in the case of convulsions, must possess, besides a cerebral fibre, also a spinal fibre.

When, now, according to what has been stated, it is extremely probable that not merely the spinal fibres, but also the cerebral fibres, are the media of reflex-functions, it certainly cannot be concluded, from the reflective power of a nerve, whether it is mixed, that is, whether it possesses both these species of fibres, or only one. The conclusion, for instance, that the trigeminus, in its expansion on the organ of vision, contains excitatory fibres, because irritation of the conjunctiva produces reflex motions of the eye-lids, is unsatisfactory, as its sensitive fibres appear not less appropriated to the production of such motions. The division of the nerves given by Dr. M. Hall accordingly rests, from beginning to end, on a weak foundation, but the development of the system is even more defective than it needed to have been according to its own principles. His division is as follows:—

1st. Class. Cerebral—or Sensomotory Nerves.

A. *Sensitive Nerves.* Olfactory, Optic, great portion of the Trigeminus, the N. Auditorius, N. Glossopharyngeus, the posterior roots of the Spinal Nerves.

B. *Spontano-motory Nerves.* N. Oculomotorius, small portion of the Trigeminus, N. Hypoglossus, the anterior roots of the Spinal Nerves.

2nd Class. Spinal or Excito-motory Nerves.

A. Excitatory Nerves, great portion of the Trigeminus, Vagus, posterior roots of the Spinal Nerves.

*a.* Excitatory Trigeminus, in its expansions on the eye-lids, alæ nasi, throat, skin of the face.

*b.* Excitatory Vagus: in the larynx, pharynx, stomach, and lungs.

*c.* Posterior Spinal Roots excitatory: at the anus, neck of the bladder, os uteri, cutaneous surface.

B. *Reflecto-motory Nerves,* (presiding over involuntary motions.) N. Patheticus, Abducens, Facialis, in its distribution to the M. Orbicularis (oris?) Vagus, to the larynx and pharynx, the Accessorius Willisii, anterior roots of the Spinal Nerves.

3rd Class. Ganglionic Nerves.

This division however appears in a great degree arbitrary, and scarcely has any one nerve received a place in it which might not be attacked in one respect or other.

To adduce a few cases, the nerves of sense are thrown exclusively into the first division of the first class, but they do not merely serve for sensation, they also excite involuntary motions, as, for instance, irritation of the nasal membrane produces contraction of the iris and closing of the eye-lids.

The vagus is ranked under the excitatory, not under the sensitive nerves, which cannot be understood otherwise than that its power to convey irritation to the brain must depend on fibres of a peculiar kind, that is, on excitatory fibres. And yet many observers, on dividing the vagus, have seen manifest signs of pain: Bischoff and Brachet have recently placed the sensitive power of this nerve in the clearest light, and when Broughton, on dividing this nerve, observed no signs of pain, the cause of it was, that the pain of the preceding operation threw the animal into that state of dumb reaction, which so often takes place in vivisections. Further, with respect to the nerves, by which motions are conducted, (not to mention the facial nerve,) the N. Patheticus, Abducens, and the nerves of the voice, are unaccountably omitted among the spontano-motory fibres; on the other hand, the oculo-motorius is only numbered among the nerves of voluntary motion, just as if it were incapable of reflex motions. However, according to Mayo, the motions of the iris depend on these nerves, and the irritation of worms in the intestines occasion dilatation of the pupils on the principle of reflexion. Lastly, that division of the nerves is so far incorrect, as it perfectly excludes the ganglionic nerves from the list, without making reference to their admixture, as well with sensitive as with excito-motory fibres. As for instance in that division, the Trigeminus, is misplaced no less in the second than in the first class, because it appears to contain excito-motory besides senso-motory fibres, the sympathetic must accordingly be mentioned in the first division of the first class among the sensitive fibres, and, in the second class, in both divisions, accordingly among the exciting and among the reflecting fibres. If the Trigeminus is to pass as a mixed nerve of second power, that is, as a nerve, in which not only sensitive fibres mix with spontano-motory fibres, but these again with excitatory and reflecting fibres; the Sympathetic then must be ranked as a mixed nerve of third power, as besides sympathetic fibres, it also contains sensitive, excitatory, and reflecting fibres.

We have now presented our readers with the principal points contained in this paper of Professor Volkmann; sufficient of it at least to shew that the reflex-function system is far from being a perfect one. Dr. Marshall Hall can hardly expect that the reflex-function, together with the apparatus by which it is performed, has yet attained anything like perfection; and we are sure that, instead of viewing with a jealous eye, the experiments of other physiologists, whether corroborative or subversive of his doctrines, he will hail them with joy. We think that experiments should be carefully and frequently performed on animals, instead of wasting words, time, and large quantities of ink, in controversies as to priority of discovery. This hint may be usefully acted on by both parties in this bellum horridum.

